# Relationship of Elongated Styloid Process in Digital Panoramic Radiography with Carotid Intima Thickness and Carotid Atheroma in Doppler Ultrasonography in Osteoporotic Females

**Published:** 2015-06

**Authors:** Shahram Hamedani, Mohammad Hossein Dabbaghmanesh, Zahra Zare, Mahvash Hasani, Mahshid Torabi Ardakani, Mahsa Hasani, Shoaleh Shahidi

**Affiliations:** 1 DDS, MSc in Dentomaxillofacial Radiology, Dental Research Development Center, School of Dentistry, Shiraz university of Medical Sciences, Shiraz, Iran;; 2 Shiraz Endocrine and Metabolism Research Center, Shiraz University of Medical Sciences, Shiraz, Iran;; 3 Dept. of Maxillofacial Radiology, School of Medicine, Shiraz University of Medical Sciences, Shiraz, Iran;; 4 Dept. of Maxillofacial Radiology, School of Dentistry, Shiraz University of Medical Sciences, Shiraz, Iran;; 5 Private dentist, Shiraz, Iran;; 6 Student Research Committee, Shiraz University of Medical Science, Shiraz, Iran;; 7 Biomaterial Research Center, Dept. of Oral and Maxillofacial Radiology, School of Dentistry, Shiraz University of Medical Sciences, Shiraz, Iran;

**Keywords:** Digital radiography, Panoramic radiography, Carotid intima thickness, Doppler ultrasonography, Carotid atheroma

## Abstract

**Statement of the Problem:**

Cardiovascular disease and osteoporosis are major health dilemmas. Osteoporotic patients frequently display vascular calcification that consequently increases the cardiovascular morbidity and mortality.

**Purpose:**

This study aimed to investigate the relation of osteoporosis, vascular calcification (atheroma, intima-media thickness (IMT)) and elongated styloid process (ESP) in a sample of osteoporotic and normal female individuals.

**Materials and Method:**

This study recruited 78 women who were assessed for bone mass density (BMD). Sample included individuals with normal BMD (n=13, 17 %), osteopenia (n=36, 46 %), and osteoporosis (n=29, 37%). The presence of atheroma and IMT was examined using color Doppler ultrasonography (CD-US). In addition, digital panoramic radiographs (PRs) were obtained to assess ESP.

**Results:**

In this study, 55 subjects (70%) with low BMD exhibited at least one side ESP. Femoral BMD decreased significantly in subjects with ESP (*p*= 0.03). Bilateral ESP was correlated with the presence of atheroma (*p*= 0.029). The CIMT was greater in patients with ESP, although the relation was not significant.

**Conclusion:**

The obtained data suggest referring the aged individuals with ESP for BMD assessment and individuals with low bone mass and ESP for more cardiovascular risk assessment.

## Introduction

Osteoporosis is a progressive skeletal disease in which the amount of bone is reduced and the trabecular architecture is modified. It is more prevalent in postmenopausal women; however, both men and women with underlying conditions would experience the bone loss.[1]

The first study about the association between osteoporosis and oral bone was conducted in 1972.[2] The manifestations of osteoporosis in dentomaxillofacial bone structures include loss of periodontal attachment,[3] loss of teeth, bone loss in the jaws, reduction in the height of alveolar bone,[4] erosion of the inferior mandibular cortex.[5]To the best of authors’ knowledge, there is only one English study regarding the relationship of osteoporosis and elongation of styloid process and presence of atheroma.[6]

Styloid process is a long cartilaginous bony projection placed on the temporal bone, just anterior to the stylomastoid foramen.[7] The average normal length of the styloid process varies from 20 to 25 mm. The styloid process is assumed to be elongated if it is longer than 30mm.[8-9]

Cardiovascular disease and osteoporosis are considered as major health problems. Epidemiological studies suggest that the underlying pathophysiologic mechanisms are similar in osteoporosis and cardiovascular disease.[10-14] Increasing in the intima-media thickness (IMT) is known to be related to the increased risk of myocardial infarction and ischemic stroke.[15] Also, it is related inversely to the lumbar spine BMD in postmenopausal women.[16] Furthermore, low bone mass is associated with echogenic carotid plaques assessed by Doppler ultrasonography.[17]

Regarding the limited studies that support the association between the osteoporosis and elongation (calcification) of the stylohyoid complex and vascular calcification,[6, 18] the current study aimed to scrutinize the presence of ESP in digital panoramic radiographs (PRs)[19] and carotid atheroma as well as IMT in CD-US in osteoporotic patients versus normal group. This study employs a gold standard method,[20] color Doppler ultrasonography (CD-US), to evaluate the vascular calcification. It will also investigate the associations between the ESP and vascular calcifications and osteoporosis.

## Materials and Method

In this study, 95 subjects were recruited from the referees to the bone densitometry center (Namazi Hospital, Iran) during April to November 2011. This study was in compliance with the Helsinki Declaration; all participants were completely informed about the details of the study and relevant consent forms were signed after proper information. 

The exclusion criteria were: 1) having unknown precise medical history; 2) tobacco or alcohol use; 3) patients with metabolic bone diseases (such as hyperparathyroidism, hypoparathyroidism); 4) use of medications that affect bone metabolism (such as corticosteroid); 5) having any disease that affects cardiovascular system (such as diabetes, hypertension, hyperlipidemia and so on).

*BMD Assessment*

BMD at the lumbar vertebrae (L2-L4), hip and neck of the femur was determined using dual energy x-ray absorptiometry (DXA, LUNAR DPX IQ). The BMD results of post-menopausal patients were expressed as standard deviations from the bone mass scores of the young female patients participated in the same study (T-score).

According to WHO criteria, they were classified as: *Normal*; with BMD ≥−1 standard deviations (SD) from young adult mean, *Osteopenia*; with BMD ranging from -1 to -2.5 SD below the young adult mean, and *Osteoporosis*; with BMD ≤-2.5 SD from the young adult mean.

*Panoramic radiographs (PRs)*

The digital panoramic radiographs were taken by employing Proline XC (Planmeca; Finland) with digital processor (Regious 110; Konica Minolta) at Dental and maxillofacial radiology department of Shiraz Dental School, Iran. The conventional protocol was used in exposing the plates with no extra modification of radiographic technique or any alteration in positioning. The radiographs were interpreted by two expert oral and maxillofacial radiologists blinded to the study. The study radiographs were viewed consecutively in a room with dimmed light.

**Figure 1:**
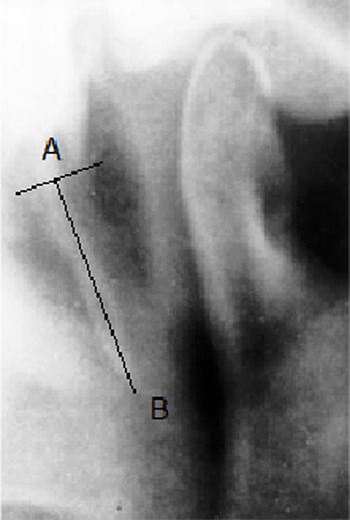
Anatomical landmark used as a reference point for assessing the length of styloid: the cleft between tympanic plate and SP, measuring the SP from this point (A) to the end (B).

The length of styloid processes from both sides was assessed through linear measurement by Digora DfW software and values higher than 30 mm were adopted as the manifestation of elongated styloid. Fifteen cases were excluded from the study since the origin of styloid process from the lower part of temporal bone was hidden by the shadows of base of the skull; hence, detection of its origin on the radiograph was difficult. In our study, styloid length evaluation was done according to the study of Jung *et al.*[21] The measurements were performed from the frontal view of the SP where it leaves the tympanic plate of the temporal bone. In this area on the PR, a thin transparent line was generally visualized between the shadows of the SP and the tympanic bone. This transparent line is corresponding to the cleft between the SP and the tympanic plate of the temporal bone (Figure 1). The starting point of measurement (A) was considered where this radiolucency was finished. This point is almost concomitant with a perpendicular line drawn from the tip of the tympanic plate to the long axis of the styloid process. The tip of the SP -the ending point of the measurement (B) - was its bony end, including mineralized parts of the ligament. The length of the line AB was considered as the length of styloid process in this study (Figure 1).

To reduce the intra-observer bias, measurements were repeated after 3 weeks for a total of 20 panoramic radiographs, randomly allocated. Deviations of the mean length of SP between the first and second measurements were 1.5%.

*Color Doppler Ultrasonography (CD-US)*

Finally, the patients were referred to radiology department of Sa’adi Hospital, Shiraz, Iran, and Carotid Doppler (β mode ultrasound) sonography was performed by an experienced medical radiologist based on a set protocol. The patient was in the supine position with the neck in slight hyperextension for an optimal visualization of the common carotid arteries and carotid bulb of both sides. The intima thickness of the carotid arteries was evaluated bilaterally with a US device (Esaote; MyLab 70, Italy) using a 10-MHz linear type-B–mode probe. The presence of atheroma was then assessed by color Doppler.          

*Statistical Analysis*

Predictive Analysis Software (PASW, (SPSS version 15)) was employed to analyze the results. The subjects were divided into 2 groups based on the BMD of the femoral, spinal and hip. The subjects with normal BMD were assigned group 0 and subjects with low BMD (osteopenia, osteoporosis) were allocated group 1.

The subjects were also divided into 2 groups based on the measurement of styloid process; normal length (0= <30mm) and elongated styloid process (1= ≥30mm). Furthermore, they were classified into 2 groups according to the presence of atheroma; 0=no atheroma, 1= presence of atheroma. Pearson’s correlation coefficient and t-test were employed to evaluate the association between the hip, femoral and spinal BMD, carotid intima-media thickness, atheroma and elongated styloid process. 

## Results

The study participants were 78 females and 2 males aged 38-65 years; the 2 men were excluded to keep the uniformity of results. The mean age of the osteoporotic, osteopenic and normal groups were 64.27, 52.35 and 45.86 years, respectively (whole mean age 54.1). The mean age of the patients with ESP and without was 54.35 versus 49.65 years. Based on the femur BMD, 35% were classified as osteoporotic, 46.2% as osteopenic and 18.8% had normal BMD. All recorded data are illustrated in Table 1.

**Table 1:** The age group and variable prevalence

**Mean (SD)**								
**Age** **group**	**Number** **of patients**	**Spine** ** Densitometry (g/cm^2^) **	**Hip** **densitometry**	**Femoral neck ** ** densitometry (g/cm^2^) **	**One** **ESP**	**Both** **ESP**	**Atheroma**	**Mean IMT** **(mm)**
31-40	2	0.770 (0.417)	0.945 (0.041)	0.918 (0.137)	1	0	0	0.445 (0.707)
41-50	32	0.879 (0.157)	0840 (0.128)	0.729 (0.116)	8	18	1	0.503 (0.165)
51-60	27	0.754 (0.124)	0.776 (0.123)	0.658 (0.119)	8	15	6	0.523 (0.096)
61-70	17	0.658 (0.162)	0.675 (0.124)	0.544 (0.090)	5	11	5	0.636 (0119)
Total	78	0.785 (0.174)	0.784 (0.140)	0.669 (0.136)	22	44	12	0.537 (0.142)

*Relationship between BMD and ESP*

In the correlation analysis, association was found between femoral BMD and the elongation of right styloid process (r=-0.243; *p*= 0.030). However, in cases where the osteoporosis was detected on all three analyzed sites (femur, spine and hip), correlation was found with elongated styloid processes (*p*> 0.05) neither on one nor on both sides. BMD scores in subjects with ESP were lower than individuals without ESP.

*Association between BMD and Atheroma*

Out of 78 subjects, 15% had atheromatous plaque and also low bone mass in the spine and femur. No subject with normal BMD had atheroma. The relationship between presence of atheroma in the left carotid artery and femoral BMD was confirmed (r=-0.238; *p*= 0.034). Also, there was a significant linear correlation between the presence of atheroma, at least in one side, and femoral neck densitometry result (r= -0.301; *p*= 0.007).

*Relationship between BMD and IMT*

The relationship between IMT and spine, hip and femur BMD was not statistically significant (*p*= 0.652). But the IMT was higher in subjects with low bone mass than normal BMD subjects.

*Relationship of ESP with Atheroma, IMT*

A total of 12 atheroma cases were detected in CD-US, 10 of which were in subjects with ESP. Presence of atheroma and ESP on one side were not correlated (*p*> 0.05). There was a significant relationship between the presence of atheroma and ESP on both sides (*p*= 0.029). IMT was greater in patients with styloid process longer than 30 mm. However, the correlation between IMT and elongated styloid process was not statistically significant (*p*> 0.05).

## Discussion

This cross-sectional study confirmed that extra-osseous bone formation,[22] namely vascular and stylohyoid calcification is prevalent in osteopenic and osteoporotic adults. Panoramic radiography, as a routine radiological evaluation in dental practices, allows practitioners to analyze components of the stomatognathic system as well as other near structures. It is possible to identify and measure the length of styloid process of the temporal bone by this technique, using the images of the external acoustic meatus and tympanic plate as the reference points.[21] Therefore, ESP can be easily detected on these radiographs.

Mineralization or ossification of the styloid process is a common finding. In analysis of digital panoramic radiographs, Chandramani and Mukesh reported 19.4% incidence of ESP.[23] The prevalence in Iranian population was reported to be 36.4% according to the study by Ghafari *et al.*[24] Calcified styloid processes were more common in patients between 50 and 69 years.[25] The rate of ESP in Iranian individuals (>40 years old) was 45.6%.[26] Our study showed that 43 of 49 patients (89%) in the age group between 51 to 65 years had at least one elongated styloid process, which is in accordance with the results of other studies.[25-27]

The first investigation on the relationship between ESP, vascular calcification and osteoporosis was carried out in 2010.[6] According to the study by Watanabe *et al.*, 80 percent of patients presented ESP in at least one side.[6] In our study, 56 individuals (70%) with diagnosed osteopenia or osteoporosis (at least in two sites) had elongated styloid process. This result might indicate a possible association between low BMD and ectopic calcification process in stylohyoid ligament.

Based on our results, ESP was presented two times more in patients with low bone mass than normal individuals. The study found a significant inverse relationship between femoral BMD score and elongated styloid process (r=-0.243; *p*= 0.030).This finding was compatible with the results obtained by Watanabe *et al.* that found an association between osteopenia/ osteoporosis diagnosed on radium, column, head of femur and the ESP.[6] By contrast, Okabe *et al.*found that there was a correlation among the length of the calcified SP, serum calcium concentration and heel bone density. They suggested that SPE may be a predictor of high BMD and high serum calcium level.[18]

Atheroma was reported from 2% to 5% in adult population, with a higher frequency rate in menopause women and in individuals aged 65 years and more.[28] A recent investigation in osteoporotic patients, using digital panoramic radiographs, demonstrated 8% incidence (four patients) of vascular calcification,[6] while an incidence of atheroma in this study was 15% (twelve patients). Employing panoramic radiograph in detection of calcification in the carotid artery, was reported to be limited and of low sensitivity.[6, 29] The current study used gold standard techniques, CD-US, for detecting atheroma which could explain the higher incidence of calcification. Another important finding in our study was the presence of all atheromatous plaques in patients with low bone mass. Our study demonstrated a significant correlation between osteopenia/osteoporosis in the femoral site and atheroma. This finding is in line with the results of previous longitudinal studies which reported the bone loss to be positively related to the progression of atheroma.[30-32] Jorgensen *et al.* demonstrated that presence of echogenic calcified carotid plaques on ultrasonography was higher in subjects with a low forearm BMD.[17] This result was also in accordance with the findings of the current study which detected atheroma in low BMD subjects.

Common carotid intima-media thickness, known to be associated with an increased risk of myocardial infarction and ischemic stroke,[15] is inversely related to the lumbar spine BMD in postmenopausal women.[16] In our study, there was not a significant relationship between IMT and BMD. Probably, this dissimilar result is due to the relatively young age ranges of our subjects (54.1 years) in comparison with the mean age of other studies (more than 65 years).[32-33] Although the literature indicates this association is regardless of age, a cut-off point for age (65 years) was also established for the association of IMD and BMD.[32-33]

The current study demonstrated that IMT in subjects with ESP was higher than the individuals without ESP (0.540 mm versus 0.479 mm right IMT).

While unilateral elongated ESP was not related to the presence of atheroma, presence of bilateral ESP and atheroma were correlated (*p*= 0.029). This result would probably reconfirm the relation among osteoporosis, atheroma and bilateral ESP.

Similar to the findings of the current study, clinical observations demonstrate coincidence of systemic osteoporosis and systemic inflammation.[34] Other studies revealed that the estrogen withdrawal can induce the bone loss through activation of osteoclasts by pro-inflammatory cytokines.[35-36] Furthermore, inflammation causes vascular calcification[12] and based on the study performed by Al-Khateeb *et al.*, inflammation instigates the mineralization of the stylohyoid complex.[37] Moreover, Hiro *et al.* reported that inflammation produces a trans-differentiation of the regional undifferentiated mesenchymal cells into osteoblasts.[38]

## Conclusion

The panoramic radiological images which can provide imperative information on general health condition of the patient can disclose the ESP (ectopic calcification) and reveal its possible relation with the presence of osteoporosis.

Based on the results yielded by this study, an association was found between ESP and osteoporosis and also between ESP and presence of atheroma. The authors would suggest referring individuals (> 40 years old) with ESP for BMD assessment. They also recommend referring the individuals with low bone mass and ESP for further cardiovascular assessment due to the potential risk of atheroma. Further studies, enrolling larger normal and osteoporotic BMD samples, are recommended. 

## References

[1] Jeddi M, Roosta MJ, Dabbaghmanesh MH, Omrani GR, Ayatollahi SM, Bagheri Z (2013). Normative data and percentile curves of bone mineral density in healthy Iranian children aged 9-18 years. Arch Osteoporos.

[2] Hildebolt CF (1997). Osteoporosis and oral bone loss. Dentomaxillofac Radiol.

[3] Von Wowern N, Klausen B, Kollerup G (1994). Osteoporosis: a risk factor in periodontal disease. J Periodontol.

[4] Southard TE, Southard KA (1996). Detection of simulated osteoporosis in maxillae using radiographic texture analysis. IEEE Trans Biomed Eng.

[5] Dervis E (2005). Oral implications of osteoporosis. Oral Surg Oral Med Oral Pathol Oral Radiol Endod.

[6] Watanabe PC, Dias FC, Issa JP, Monteiro SA, de Paula FJ, Tiossi R (2010). Elongated styloid process and atheroma in panoramic radiography and its relationship with systemic osteoporosis and osteopenia. Osteoporos Int.

[7] Neville BW, Damm DD, Allen CM, Bouquot JE (1997). Developmental defects of the oral and maxillofacial region.

[8] Gokce C, Sisman Y, Ertas ET, Akgunlu F, Ozturk A (2008). Prevalence of styloid process elongation on panoramic radiography in the Turkey population from cappadocia region. Eur J Dent.

[9] Kursoglu P, Unalan F, Erdem T (2005). Radiological evaluation of the styloid process in young adults resident in Turkey's Yeditepe University faculty of dentistry. Oral Surg Oral Med Oral Pathol Oral Radiol Endod.

[10] Kiel DP, Kauppila LI, Cupples LA, Hannan MT, O'Donnell CJ, Wilson PW (2001). Bone loss and the progression of abdominal aortic calcification over a 25 year period: the Framingham Heart Study. Calcif Tissue Int.

[11] McFarlane SI, Muniyappa R, Shin JJ, Bahtiyar G, Sowers JR (2004). Osteoporosis and cardiovascular disease: brittle bones and boned arteries, is there a link?. Endocrine.

[12] Farhat GN, Cauley JA, Matthews KA, Newman AB, Johnston J, Mackey R (2006). Volumetric BMD and vascular calcification in middle-aged women: the Study of Women's Health Across the Nation. J Bone Miner Res.

[13] Farhat GN, Strotmeyer ES, Newman AB, Sutton Tyrrell K, Bauer DC, Harris T (2006). Volumetric and areal bone mineral density measures are associated with cardiovascular disease in older men and women: the health, aging, and body composition study. Calcif Tissue Int.

[14] Frost ML, Grella R, Millasseau SC, Jiang BY, Hampson G, Fogelman I, Chowienczyk PJ (2008). Relationship of calcification of atherosclerotic plaque and arterial stiffness to bone mineral density and osteoprotegerin in postmenopausal women referred for osteoporosis screening. Calcif Tissue Int.

[15] De Groot E, van Leuven SI, Duivenvoorden R, Meuwese MC, Akdim F, Bots ML (2008). Measurement of carotid intima-media thickness to assess progression and regression of atherosclerosis. Nat Clin Pract Cardiovasc Med.

[16] Sumino H, Ichikawa S, Kasama S, Takahashi T, Sakamoto H, Kumakura H (2008). Relationship between carotid atherosclerosis and lumbar spine bone mineral density in postmenopausal women. Hypertens Res.

[17] Jørgensen L, Joakimsen O, Rosvold Berntsen GK, Heuch I, Jacobsen BK (2004). Low bone mineral density is related to echogenic carotid artery plaques: a population-based study. Am J Epidemiol.

[18] Okabe S, Morimoto Y, Ansai T, Yamada K, Tanaka T, Awano S (2006). Clinical significance and variation of the advanced calcified stylohyoid complex detected by panoramic radiographs among 80-year-old subjects. Dentomaxillofac Radiol.

[19] Khojastehpour L, Shahidi Sh, Barghan S, Aflaki EL (2009). Efficacy of Panoramic Mandibular Index in Diagnosing Osteoporosis in Women. J Dent Tehran Univ Med Scien.

[20] Ertas ET, Sisman Y (2011). Detection of incidental carotid artery calcifications during dental examinations: panoramic radiography as an important aid in dentistry. Oral Surg Oral Med Oral Pathol Oral Radiol Endod.

[21] Jung T, Tschernitschek H, Hippen H, Schneider B, Borchers L (2004). Elongated styloid process: when is it really elongated?. Dentomaxillofac Radiol.

[22] Karasik D, Kiel DP, Kiely DK, Cupples LA, Wilson PW, O'Donnell CJ (2006). Abdominal aortic calcification and exostoses at the hand and lumbar spine: the Framingham Study. Calcif Tissue Int.

[23] More CB, Asrani MK (2010). Evaluation of the styloid process on digital panoramic radiographs. Indian J Radiol Imaging.

[24] Ghafari R, Dalili Z, Abdolahpur S (2010). A study on the frequency of elongated styloid process and eagle’s syndrome among patients admitted to Guilan dental school clinic (2005-2006). J Isfahan Dent Sch.

[25] Rizzatti Barbosa CM, Ribeiro MC, Silva Concilio LR, Di Hipolito O, Ambrosano GM (2005). Is an elongated stylohyoid process prevalent in the elderly? A radiographic study in a Brazilian population. Gerodontology.

[26] Ghafari R, Hosseini B, Shirani AM, Manochehrifar H, Saghaie S (2012). Relationship between the elongated styloid process in panoramic radiographs and some of the general health conditions in patients over 40 years of age in the Iranian population. Dent Res J (Isfahan).

[27] Gulsahi A, Yüzügüllü B, Imirzalioglu P, Genç Y (2008). Assessment of panoramic radiomorphometric indices in Turkish patients of different age groups, gender and dental status. Dentomaxillofac Radiol.

[28] Madden RP, Hodges JS, Salmen CW, Rindal DB, Tunio J, Michalowicz BS (2007). Utility of panoramic radiographs in detecting cervical calcified carotid atheroma. Oral Surg Oral Med Oral Pathol Oral Radiol Endod.

[29] Tankò LB, Bagger YZ, Christiansen C (2003). Low bone mineral density in the hip as a marker of advanced atherosclerosis in elderly women. Calcif Tissue Int.

[30] Montalcini T, Emanuele V, Ceravolo R, Gorgone G, Sesti G, Perticone F (2004). Relation of low bone mineral density and carotid atherosclerosis in postmenopausal women. Am J Cardiol.

[31] Tankó LB, Christiansen C, Cox DA, Geiger MJ, McNabb MA, Cummings SR (2005). Relationship between osteoporosis and cardiovascular disease in postmenopausal women. J Bone Miner Res.

[32] Tamaki J, Iki M, Hirano Y, Sato Y, Kajita E, Kagamimori S (2009). Low bone mass is associated with carotid atherosclerosis in postmenopausal women: the Japanese Population-based Osteoporosis (JPOS) Cohort Study. Osteoporos Int.

[33] Reddy J, Bilezikian JP, Smith SJ, Mosca L (2008). Reduced bone mineral density is associated with breast arterial calcification. J Clin Endocrinol Metab.

[34] Yun AJ, Lee PY (2004). Maldaptation of the link between inflammation and bone turnover may be a key determinant of osteoporosis. Med Hypotheses.

[35] Gregory R, Mundy MD (2007). Osteoporosis and Inflammation. Nutrition Reviews.

[36] Anderson GL, Limacher M, Assaf AR, Bassford T, Beresford SA, Black H (2004). Effects of conjugated equine estrogen in postmenopausal women with hysterectomy: the Women's Health Initiative randomized controlled trial. JAMA.

[37] Al Khateeb TH, al Dajani TM, Al Jamal GA (2010). Mineralization of the stylohyoid ligament complex in a Jordanian sample: a clinicoradiographic study. J Oral Maxillofac Surg.

[38] Hirao M, Tamai N, Tsumaki N, Yoshikawa H, Myoui A (2006). Oxygen tension regulates chondrocyte differentiation and function during endochondral ossification. J Biol Chem.

